# Proteomics reveals the preliminary physiological states of the spotted seal (*Phoca largha*) pups

**DOI:** 10.1038/s41598-020-75759-2

**Published:** 2020-10-30

**Authors:** Jiashen Tian, Jing Du, Jiabo Han, Xiangbo Bao, Xinran Song, Zhichuang Lu

**Affiliations:** 1 Dalian Key Laboratory of Conservation Biology for Endangered Marine Mammals, Liaoning Ocean and Fisheries Science Research Institute, 50 Heishijiao Street, Shahekou District, Dalian, 116023 China; 2 Dalian Sun Asia Tourism Holding Co., Ltd., 608-6-8 Zhongshan Road, Shahekou District, Dalian, 116023 China

**Keywords:** Proteomic analysis, Animal physiology

## Abstract

Spotted seal (*Phoca largha*) is a critically endangered pinniped in China and South Korea. The conventional method to protect and maintain the *P. largha* population is to keep them captive in artificially controlled environments. However, little is known about the physiological differences between wild and captive *P. largha*. To generate a preliminary protein expression profile for *P. largha*, whole blood from wild and captive pups were subjected to a label-free comparative proteomic analysis. According to the results, 972 proteins were identified and predicted to perform functions related to various metabolic, immune, and cellular processes. Among the identified proteins, the expression level of 51 were significantly different between wild and captive *P. large* pups. These differentially expressed proteins were enriched in a wide range of cellular functions, including cytoskeleton, phagocytosis, proteolysis, the regulation of gene expression, and carbohydrate metabolism. The abundances of proteins involved in phagocytosis and ubiquitin-mediated proteolysis were significantly higher in the whole blood of wild *P. largha* pups than in captive individuals. In addition, heat shock protein 90-beta, were determined as the key protein associated with the differences in the wild and captive *P. largha* pups due to the most interactions of it with various differentially expressed proteins. Moreover, wild *P. largha* pups could be more nutritionally stressed and have more powerful immune capacities than captive pups. This study provides the first data on the protein composition of *P. largha* and provides useful information on the physiological characteristics for research in this species.

## Introduction

Spotted seals (*Phoca largha*) are small-bodied pinnipeds that are generally distributed in the cold sea area of the North Pacific Ocean. *P. largha* has been listed as one of the most endangered species in China and South Korea due to the destruction of their habitat by anthropogenic impacts^1^. For many wild animals, captivity in artificially-controlled environments is one of the most effective ways to ensure their conservation. During the past decades, China has continuously implemented artificial breeding and rearing activities for the maintenance of the *P. largha* population. At present, more than 1000 spotted seals are in captivity in the aquarium of China, accounting for 50% of the total population^2^. Despite advances in species conservation, captivity has also been shown to affect genetic and physiological characteristics in a variety of animals. For example, significant differences in genetics and morphology were observed between wild and captive Leon Springs pupfish, *Cyprinodon bovines*^3^. In addition, wild Indian leopards, *Panthera pardus fascia,* showed higher nucleotide diversity and amino acid polymorphisms in major histocompatibility complex genes and proteins compared to captive individuals^4^. Moreover, differences in the concentration of plasma cortisol between captive and wild harbor seals (*P. vitulina)* of the same sex and during the same season were highly significant^5^. Nonetheless, no research has been performed to describe the potential physiological differences between wild and captive spotted seals.

Previous investigations in spotted seals mainly focused on their distribution^6,7^, development^8^, and genetic diversity^9,10^. To date, only a few studies explored the physiological characteristics of spotted seals at the molecular level. Gao et al.^1^ assembled the transcriptome in liver and spleen of spotted seals and identified 193 unigenes associated with defense mechanisms. In addition, the normal levels of hematology and serum biochemistry indices in the captive spotted seals were measured, and the age- and gender-related differences in those indices were obtained^11^. Moreover, the relationships between sexual maturation and the concentrations of serum testosterone, progesterone, and estradiol in captive spotted seals were also reported^12^. The physiological functions of all organisms are achieved through the “gene-mRNA-protein” pathway. Studies at the gene and mRNA levels do not completely reflect the physiological functions of organisms due to pre- and post-transcriptional regulation^13^. Proteins are the direct performers of biological functions, and thus, measuring protein expression profiles is a powerful way to understand the physiological characteristics of spotted seals.

Proteomics technologies evaluate the complete protein composition expressed by a genome, cell or tissue, and provide powerful tools to examine the physiological functions of animals^14^. Over the years, qualitative proteomics techniques based on mass spectrometry (MS), such as 2D-gel-MS, have developed into the most direct and accurate methods for identifying the proteins in animal samples^15^. However, such traditional techniques have many shortcomings, including their inability to quantitatively recognize the differentially expressed proteins (DEPs) and their poor detection of low-abundance proteins. Hence, a quantitative proteomics technology, named label-free shotgun proteomics, was developed to determine DEPs with extreme accuracy, sensitivity, discrimination, and high-throughput^16^. At present, label-free shotgun proteomics has been widely used in humans^17^, plants^18^, and microorganisms^19^. Therefore, comparative proteomics research based on label-free shotgun proteomics is suitable for a more comprehensive comparison of the physiological functions between wild and captive spotted seals.

In the present study, preliminary whole blood protein expression profiles for wild and captive *P. largha* pups were determined using the label-free shotgun proteomics technology. The objectives of this study were to (1) describe the preliminary whole blood protein composition patterns of *P. largha* pups; (2) provide an overview of the differences in the whole blood proteomes between wild and captive *P. largha* pups; and (3) identify the key proteins that may potentially alter the physiological functions of *P. largha* pups due to captivity. To the best of our knowledge, this study is the first application of proteomics technology for the evaluation of spotted seals.

## Results

**Figure 1:**
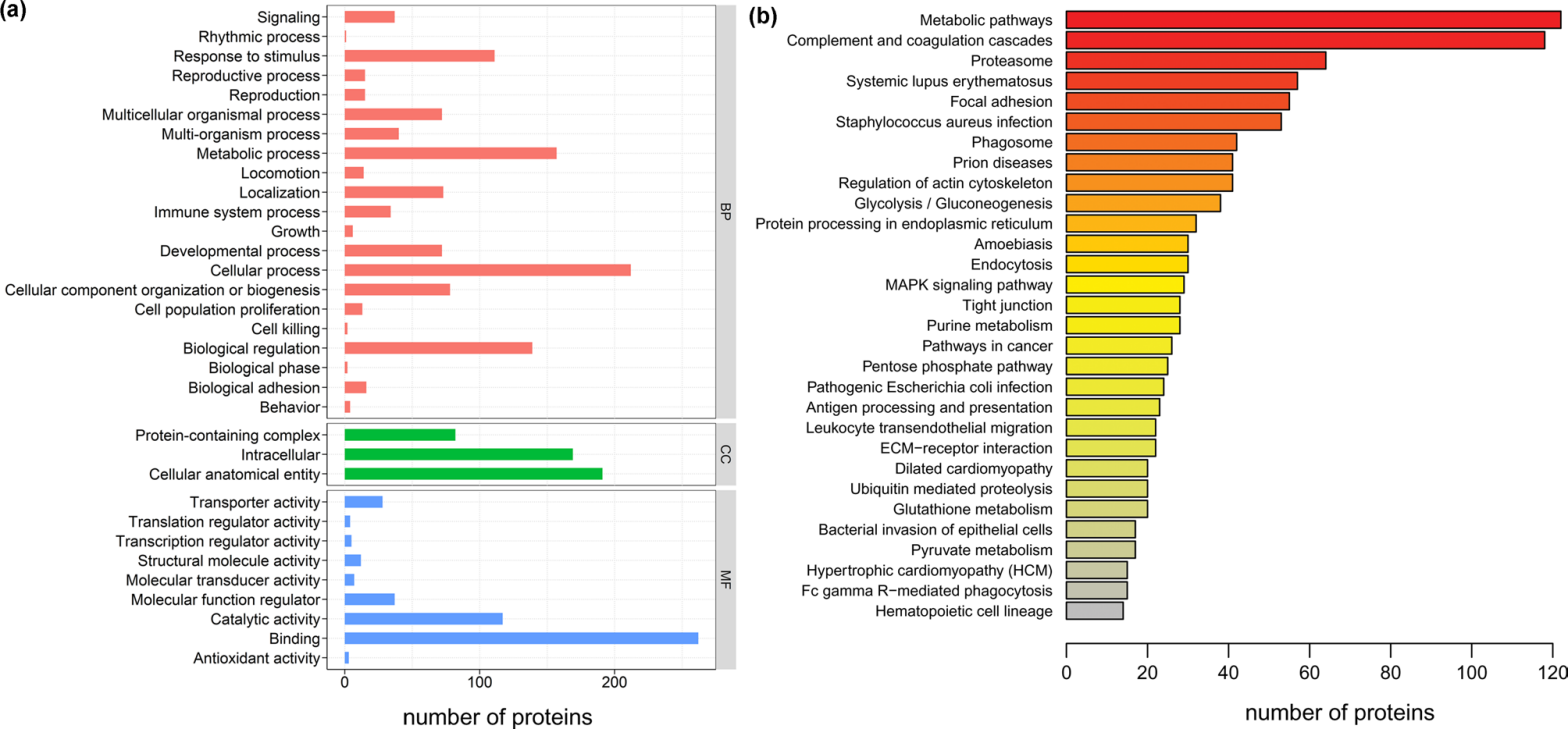
Functional distribution of the proteins identified in the whole blood of wild and captive *Phoca largha* pups based on the GO (**a**) and KEGG (**b**) databases.

A total of six whole blood samples from *P. largha* pups (three from wild pups and three from captive pups) were measured using label-free proteomic analysis. After data filtering and protein identification, a total of 4562 unique peptides were obtained and 972 proteins were identified from *P. largha* pup blood (Supplementary file 1). To investigate the physiological functions of *P. largha* pups, annotations from the Gene Ontology (GO) and Kyoto Encyclopedia of Genes and Genomes (KEGG) databases were extracted based on the reference transcripts that matched the identified *P. largha* proteins. According to the results of GO annotations, the major protein functions included cellular process, metabolic process, biological regulation, and response to stimulus belonging to the biological process aspect, intracellular and cellular anatomical entity in the cellular component aspect, and binding and catalytic activity attribute to the molecular function aspect (Fig. 1a). For KEGG pathway analyses, the identified proteins exhibited a broad functional distribution, among which the dominant pathways were metabolic pathways, and the complement and coagulation cascades (Fig. 1b).

**Figure 2:**
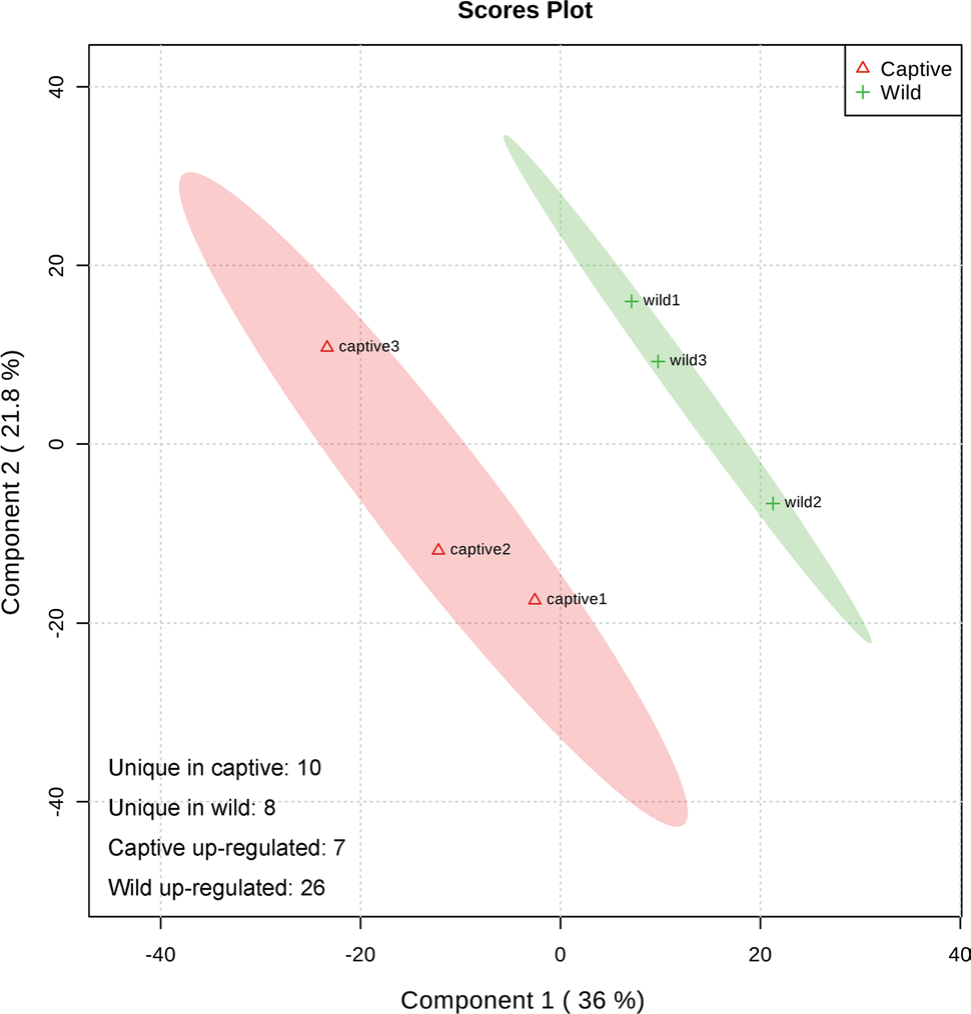
PLS-DA of proteins in the whole blood from wild and captive *Phoca largha* pups.

Based on the identified proteins, partial least squares discrimination analysis (PLS-DA) was used to comprehensively investigate the divergence of the preliminary protein expression profiles between wild and captive *P. largha* pups (Fig. 2). The first two components explained 57.8% of the total protein expression variations, and samples from the wild and captive individuals were observed in isolated clusters, separated from one another. Those results suggested that captivity in artificial environments could significantly affect the protein composition and abundance in the whole blood of *P. largha* pups. Furthermore, the expression of 51 proteins exhibited significant variations between the whole blood of wild and captive *P. largha* pups (Supplementary File 2). Compared to the samples from captive pups, the number of up-regulated DEPs in the wild-pup samples was 26 (Table 1), while seven DEPs were down-regulated (up-regulated in captive, Table 2). Moreover, there were eight and ten proteins unique to the wild- and captive-pup samples, respectively (Table 3). Thus, those results revealed the dissimilarity in the protein expression profiles in whole blood from wild and captive *P. largha* pups.

**Table 1:** Up-regulated proteins in the whole blood of wild *Phoca largha* pups compared to captive pups.

Protein identity	Fold change	P-value
Filamin A (FLNA)	9.48	0.001
Tubulin beta chain (TUBB)	6.76	0.006
Cathepsin S (CTSS)	4.22	0.039
Ubiquitin-conjugating enzyme E2-230K (UBE2)	3.59	0.024
Barrier to autointegration factor 1 (BANF1)	2.90	0.045
Alpha-2-HS-glycoprotein (AHSG)	2.78	0.034
Regulator of G-protein signaling 10 (RGS10)	2.56	0.019
Vinculin (VCL)	2.54	0.027
Ubiquitin-conjugating enzyme E2 O (UBE2O)	2.52	0.026
Plasma kallikrein (KLKB1)	2.34	0.039
NIF3-like protein 1 (NIF3L1)	2.26	0.011
S-100P (S100P)	2.26	0.025
Nucleosome assembly protein 1 (NAP1)	2.13	0.026
Protein phosphatase 1A (PP1A)	2.07	0.033
CD59 glycoprotein (CD59)	1.98	0.009
Protein phosphatase type 2A (PP2A)	1.89	0.006
Phosphatidylcholine-sterol acyltransferase precursor (LCAT)	1.86	0.025
Glucose-6-phosphate 1-dehydrogenase X (G6PDX)	1.84	0.042
Heat shock protein HSP 90-beta (HSP90AB1)	1.80	0.034
Ras suppressor protein 1 (RSU1)	1.68	0.035
Ubiquitin-like-conjugating enzyme (ATG3)	1.68	0.021
Calpain-1 catalytic subunit (CAPN1)	1.68	0.027
Calcium-regulated heat stable protein 1 (CARHSP1)	1.63	0.043
Ubiquitin-like modifier-activating enzyme 1 (UBA1)	1.62	0.048
Eukaryotic translation initiation factor 5 (EIF5)	1.60	0.035
Exportin-1 (XPO1)	1.55	0.039

**Table 2:** Down-regulated proteins in the whole blood of wild *Phoca largha* pups compared to captive pups.

Protein identity	Fold change	P-value
Adenosylhomocysteinase (AHCY)	0.65	0.040
Proteasome subunit alpha type-2 (PSMA2)	0.62	0.031
Protein DDI1 homolog 2 (DDI2)	0.62	0.002
C10C5.4	0.53	0.038
Aldehyde dehydrogenase family 1 member A3 (ALDH1A3)	0.45	0.030
Vitamin K-dependent protein S (PROS1)	0.37	0.032
Glutathione S-transferase theta-1 (GSTT1)	0.32	0.037

**Table 3:** Unique proteins in the whole blood of wild and captive *Phoca largha* pups.

Unique in wild spotted seal	Unique in captive spotted seal
60S acidic ribosomal protein P1 (RPLP1)	Aldo–keto reductase family 7 (AKR7)
Ankyrin-3 (ANK3)	Creatine kinase B (CKB)
LZIC-like isoform 2 (LZIC2)	Fibrinogen alpha chain (FGA)
Methylosome protein 50 (MEP50)	Flotillin-1 (FLOT1)
Translationally-controlled tumor protein 1 (TPT1)	Galectin-3-binding protein (LGALS3BP)
Protein argonaute-2 (AGO2)	Immunoglobulin alpha heavy chain (IGHA)
F-actin-capping protein subunit alpha-1 (CAPZA1)	Kell blood group glycoprotein (KEL)
Vasodilator-stimulated phosphoprotein (VASP)	Kynureninase (KYNU)
	Myc box-dependent-interacting protein 1 (BIN1)
	Tumor protein D54 (TPD54)

As illustrated in the DEP tables, some important immune-related proteins, such as Cathepsin S (CTSS), Protein phosphatase 1A (PP1A), CD59 glycolicprotein (CD59), Protein phosphatase type 2A (PP2A), Heat shock protein HSP 90-beta (HSP90AB1), and Calpain-1 catalytic subunit (CAPN1) were up-regulated in the blood of wild *P. largha*. In contrast, five proteins related to immune response were up-regulated or uniquely expressed in captive pups, including Glutathione S-transferase theta-1 (GSTT1), Vitamin K-dependent protein S (PROS1), Protein DDI1 homolog 2 (DDI2), Immunoglobulin alpha heavy chain (IGHA), and Galectin-3-binding protein (LGALS3BP). Moreover, several proteins involved in the regulation of gene expression were significantly up-regulated in the blood of wild *P. largha* pups, including Barrier to autointegration factor 1 (BANF1), NIF3-like protein 1 (NIF3L1), Calcium-regulated heat stable protein 1 (CARHSP1), Eukaryotic translation initiation factor 5 (EIF5), and Exportin-1 (XPO1). It is also worth noting that the expressions of multiple proteins related to cell adhesion were differentially expressed in the blood of wild *P. largha* pups, including Filamin A (FLNA), Tubulin beta chain (TUBB), Vinculin (VCL), Ankyrin-3 (ANK3), Translationally-controlled tumor protein 1 (TPT1), Protein argonaute-2 (AGO2), F-actin-capping protein (CAPZA1), Vasodilator-stimulated phosphoprotein (VASP), and Fibrinogen alpha chain (FGA).

**Figure 3:**
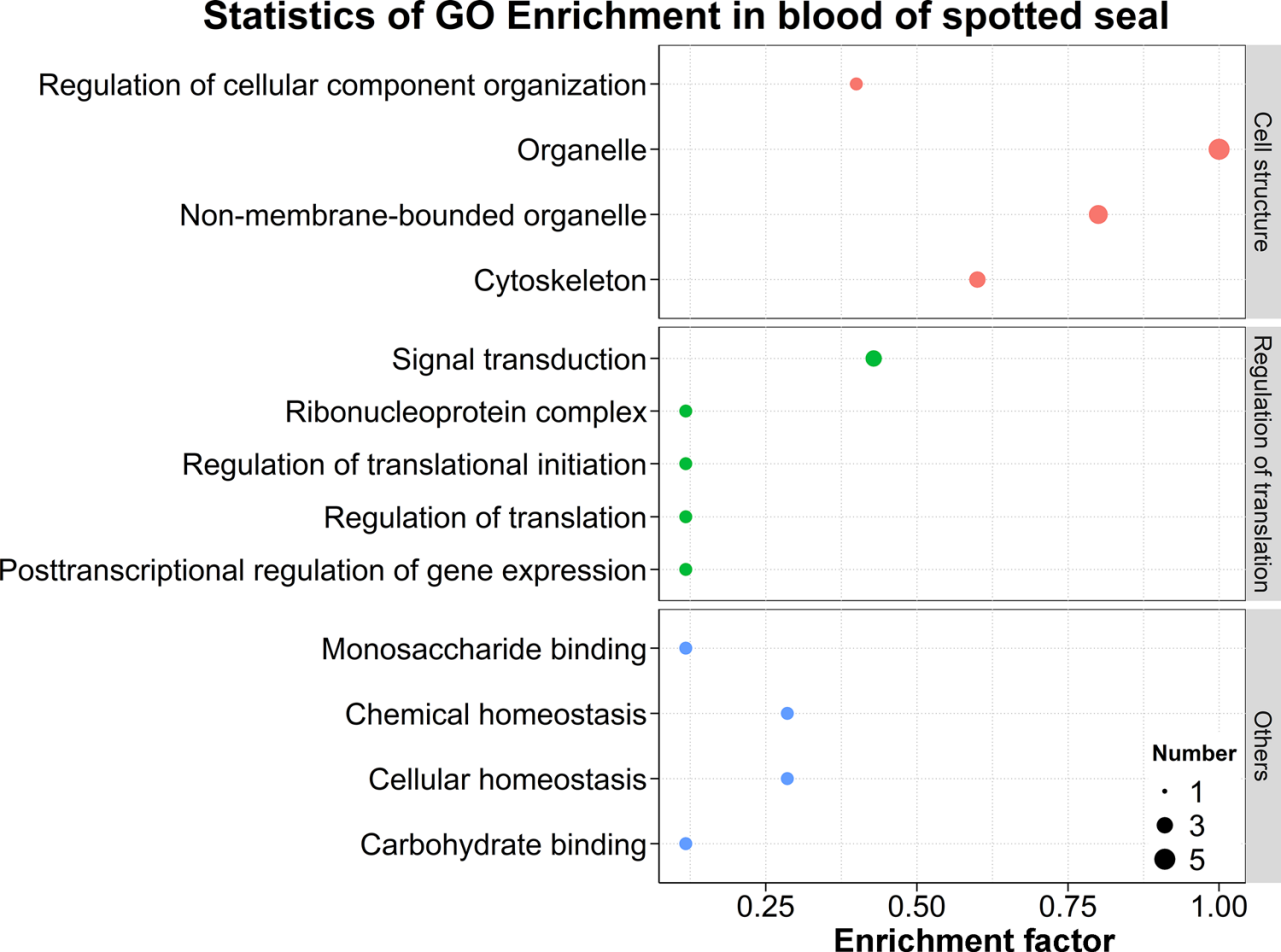
GO terms were significantly enriched (*p* adjusted by FDR < 0.05, performed by DAVID) in the whole blood of wild and captive *Phoca largha* pups based on the DEP dataset.

We also performed GO and KEGG enrichment analyses to explore the biological functions and pathways that could be significantly impacted due to captivity. The results showed that 13 GO terms were significantly enriched in the DEPs between wild and captive *P. largha* pups (Fig. 3). The enriched GO terms were assigned to three classes: (1) cell structure, including different kinds of organelle and cytoskeleton functions; (2) regulation of translation, which involves a broad range of post transcriptional and translational regulations; and (3) others, containing monosaccharide and carbohydrate binding, and cellular and chemical homeostasis.

**Figure 4:**
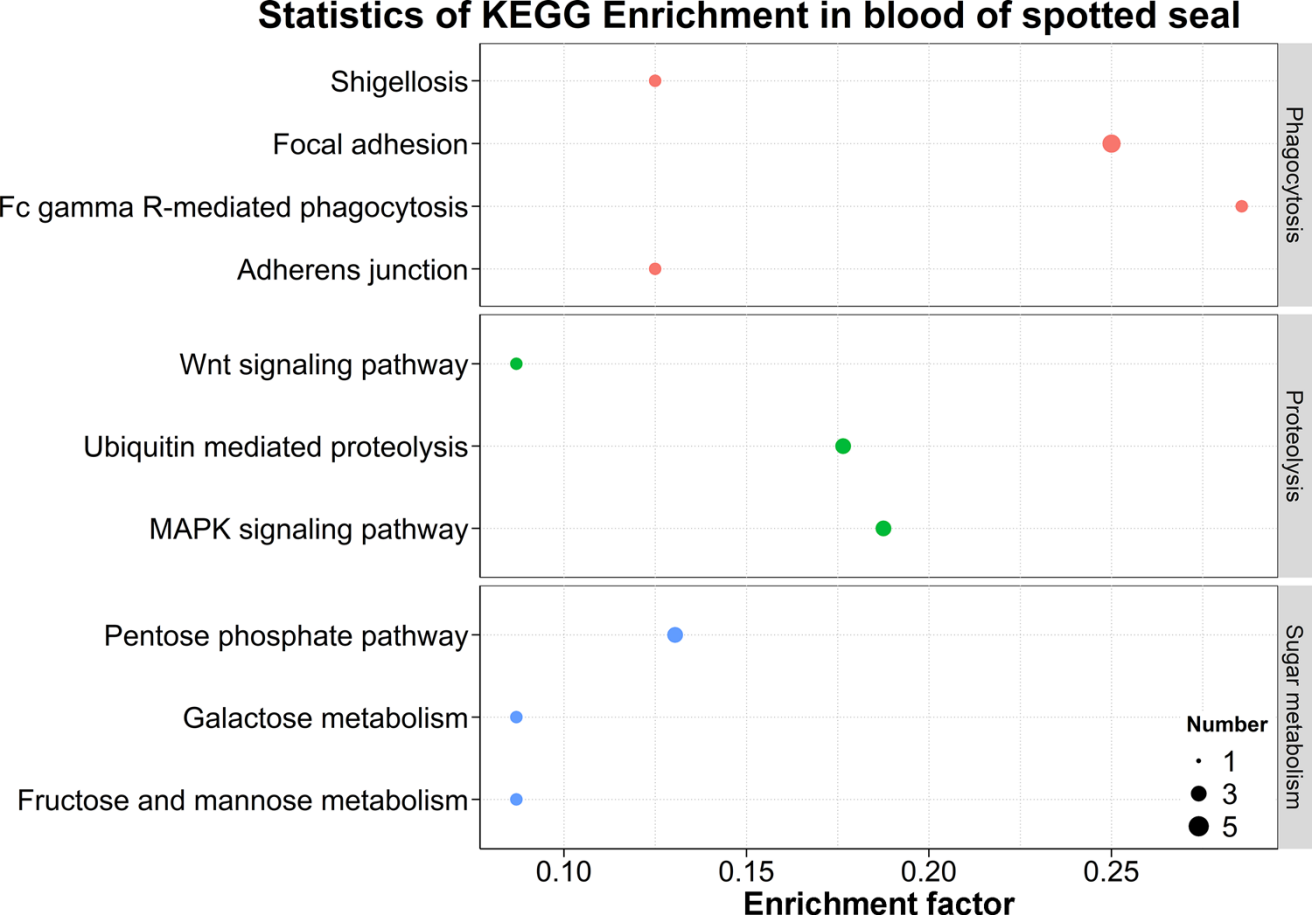
KEGG pathways were significantly enriched (*p* adjusted by FDR < 0.05, performed by DAVID) in the whole blood of wild and captive *Phoca largha* pups based on the DEP dataset.

KEGG pathway enrichment analyses based on the DEPs revealed that ten pathways were significantly enriched in the blood of wild and captive *P. largha* pups (Fig. 4). According to the function of the enriched pathways, they were classified into three categories, including phagocytosis, proteolysis, and carbohydrate metabolism. The phagocytosis category included Fc gamma R-mediated phagocytosis and its related cell adhesion pathways. The proteolysis category included ubiquitin-mediated proteolysis and its accompanying signaling pathways. The carbohydrate metabolism category included the pentose, galactose, fructose and mannose metabolism pathways.

**Figure 5:**
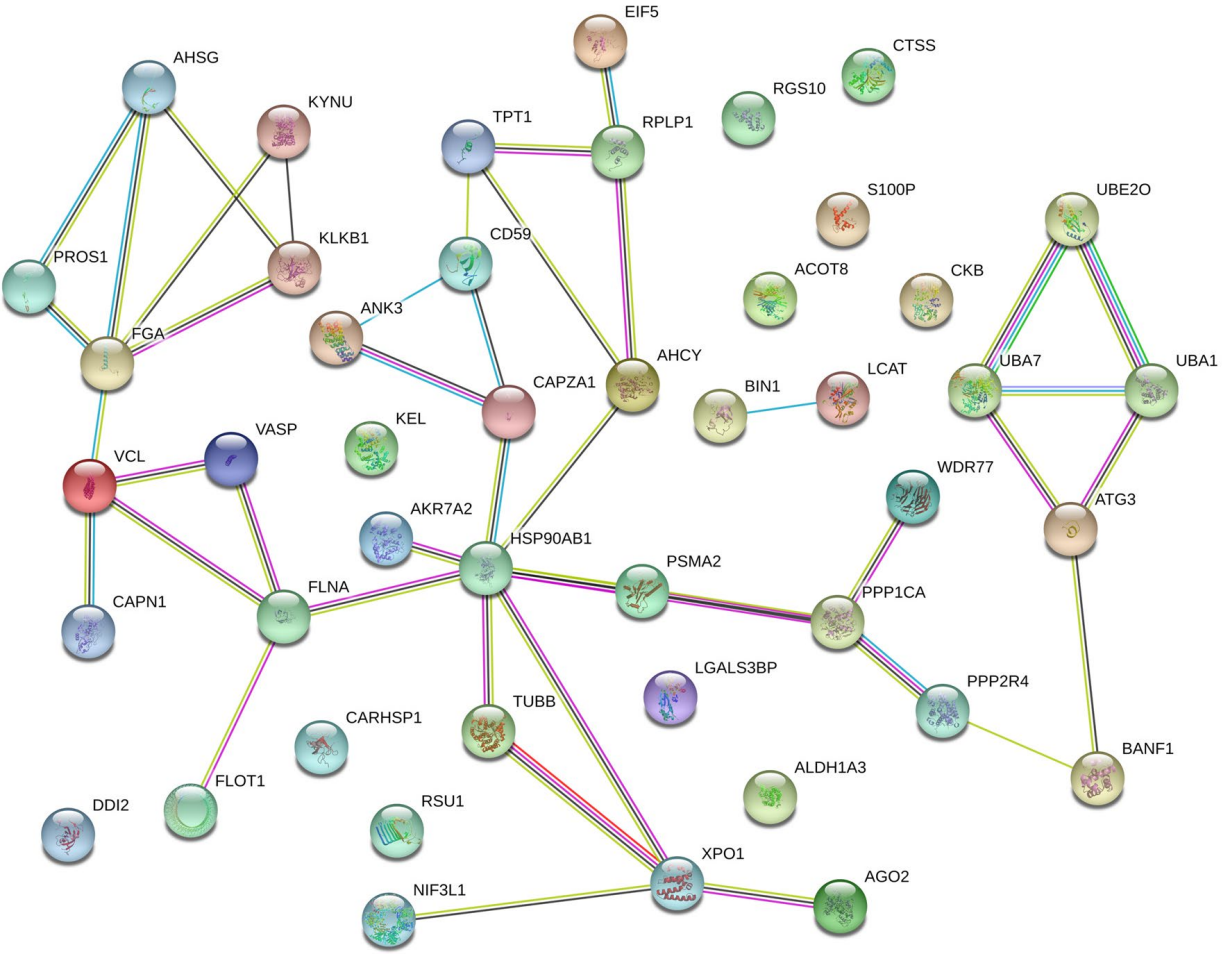
Protein–protein interaction (PPI) networks for differentially-expressed proteins between the whole blood of wild and captive *Phoca largha* pups. The PPI network had an average node degree of 1.35 and clustering coefficient of 0.491. The PPI enrichment p-value was 0.00532, which indicated that the network had significantly more interactions than expected. The colors of edges specify different types of interaction evidences and the thickness indicates the confidence of data support. STRING^20^ (https://string-db.org/) was used to construct the PPI networks of the DEPs to identify the key proteins that changed the physiological functions of *P. largha* due to captivity.

The protein–protein interaction (PPI) networks of the identified DEPs were analyzed using STRING software and the results are shown in Fig. 5. Based on the search results, HSP90AB1 had the most predicted interactions with the other DEPs. A variety of cell adhesion and cytoskeleton proteins (VASP, VCL, and FGA, among others) was predicted to interact with HSP90AB1 via FLNA. Additionally, HSP90AB1 was predicted to associate with a series of post-transcriptional (XPO1, AGO2 and NIF3L1) and translational regulation (RPLP1, EIF5, TPT1, etc.) proteins through its interactions with AHCY and TUBB, respectively. Moreover, the interaction between HSP90AB1 and PSMA2 was associated with proteins involved in ubiquitin-mediated proteolysis (UBE2O, UBA1 and UBA7).

## Discussion

Until now, no studies have been performed on the variations in protein composition and expression in *P. largha*. One of the aims of the current study was to determine the preliminary proteomic profile of *P. largha*. A total of 972 proteins were identified using label-free proteomics analyses. Regarding other marine mammals, a previous study of kidney diseases in wild animals identified a total of 2694 proteins in the urine of 19 California sea lions (*Zalophus californianus*)^21^. However, only 206 proteins were identified from the cerebrospinal fluid of 11 California sea lions^22^. Conversely, two previous studies of plasma samples from bottlenose dolphins (T*ursiops truncates*) only identified 58 and 196 unique proteins, respectively^23,24^. Thus, those results suggested that the complexity of the protein compositions of marine mammals varies between different tissues and species.

Another aim of this study was to identify the proteins that were differentially expressed between the whole blood of wild and captive *P. largha*. In the present study, the Fc gamma R-mediated phagocytosis pathway was enriched in the blood of wild and captive *P. largha* pups. Phagocytosis is an important innate immune defense mechanism in animals, and is accompanied by a chain of cellular processes as diverse as cytoskeletal rearrangement, alterations in membrane trafficking, and the activation of microbial killing mechanisms^25^.

Several cytoskeleton-related proteins were found to be up-regulated in the blood of wild *P. largha* pups compared to captive pups, including VASP, FLNA, TUBB, VCL, and CAPZA1. VASP is an actin-associated protein that promotes actin filament elongation^26^. The activation of Fc gamma receptors during phagocytosis triggers the recruitment of VASP to phagosomes^27^. FLNA is an actin-binding protein that anchors various transmembrane proteins to the actin cytoskeleton to promote cell–cell contact^28^. FLNA has been reported to play a role in neutrophil phagocytosis in humans^29^, and the deletion of FLNA resulted in a twofold reduction in collagen phagocytosis in mice^30^. The rate-limiting step during phagocytosis is the binding of specific adhesion receptors, such as integrins^31^. TUBB and VCL are intracellular adaptor proteins that link actin filaments and integrins to construct the cytoskeleton^32,33^. CAPZA1 performed to the fast-growing ends of actin filaments and its mutation resulted in actin accumulation, thereby blocking cytoskeletal rearrangement^34^. Conversely, FGA, an extracellular protein that interacts with integrins for cell–cell adhesion^35^, was uniquely detected in the blood of captive *P. largha*. It has been shown that the binding of FGA by *Streptococcus progenies* was involved in their resistance to phagocytosis in human blood^36^. Thus, all of the above findings suggest that the blood of wild *P. largha* pups contained more phagosomes than that of captive pups.

Moreover, during phagocytosis, macrophages alter the plasma membrane to form the phagosome, which is the principal site for killing microorganisms through proteolysis^37^. In the present study, proteins involved in the ubiquitin-mediated proteolysis pathway were enriched in the blood of *P. largha* pups, including UBE2, UBE2O, UBA1 and ATG3. The activation of ubiquitin-mediated proteolysis requires ATP to degrade microbial membrane proteins^38^. G6PDX, which catalyzes the rate-limiting step of the oxidative phosphate pathway to produce ATP^39^, was up-regulated in the blood of wild *P. largha* pups.

In summary, the DEPs involved in phagocytosis and proteolytic activities detected in the present study suggest that wild *P. largha* pups may be more resistant to pathogen infection than captive pups. The heightened level of immunity may be due to the result of ontogenetic differences between the two groups. In a species with such rapid post-natal development, animals that are < 1 month of age (wild pups in this study) are going to have dramatically different proteome profiles than animals that are 4 months of age (captive pups in this study). Similar to humans, newborn pups will acquire more powerful protection via maternal passive immunity from their mothers to ensure their survival^40^, and the immunity inherited from the mother could not have been completely degraded in the detected wild pups. Moreover, captive pups lived in relatively stable environments and were cared for by humans; thus, they were likely to be less environmentally stressed than wild pups. Such factors could be additional reasons for the higher expression of proteins associated with immune function of wild *P. largha* pups compared to their captive counterparts.

The biological roles of the DEPs identified in the present study were further examined based on their PPI networks to understand differences between wild and captive *P. largha* pups. HSP90AB1, a type of heat shock protein was upregulated in the wild *P. largha* pups, and was predicted to interact with a variety of DEPs of different functions, including those involved in cell adhesion, regulation of gene expression, and proteolysis. This result illustrated that HSP90AB1 could be the key protein related to the differences in the protein expression profiles between wild and captive *P. largha* pups. The realization of HSP90AB1 physiological functions depends on the consumption of ATP. Upon ATP binding, HSP90 undergoes significant conformational changes to become active and the ATP was hydrolyzed to ADP after the activation of client proteins^41^. This phenomenon was consistent with the up-regulation of proteins involved in carbohydrate metabolism in the blood of wild *P. largha* pups; thus, indicating that there might be more energy demand. Spotted seal pups in the wild are born in pack ice, which exposes them to higher predation than other animals. The pack ice breeding strategy resulted in the evolution of shorter lactation times and higher daily energy outputs in spotted seals^42^. In captive environments, newborn spotted seal pups are weaned and regularly fed. However, even if they have begun foraging, wild pups are likely to be more nutritionally-stressed than captive pups. On the other hand, wild pups are likely to respond differently to presence of and handling by researchers than pups in captivity. Sampling may induce a more robust physiological stress response in wild pups than captive pups, resulting the alteration of protein expression. Thus, the potentially higher nutritional and physiological stresses could be the underlying reason for the upregulation of proteins involved in carbohydrate metabolism and immunity in the blood of wild *P. largha* pups.

Due to ecological destruction and poaching, the number of spotted seals in Liaodng Bay, China is very low. Field rescue and captive breeding are the dominant programs for maintaining the Liaodong Bay population of spotted seals. The simultaneous occurrence of on-ice births, weaning pups, and ice-melting are important characteristics for the reproductive biology of these seals^43^. Increased ice melting due to global warming and harsh sea conditions have caused high death rates among spotted seal pups^42^. Therefore, higher expression of proteins associated with carbohydrate metabolism in wild compared to captive pups may be the result of higher metabolic demands of postnatal development in wild seals. Conversely, spotted seal pups born in aquariums are susceptible to diarrhea and pneumonia, both of which are caused by pathogenic bacteria^44,45^. The diseases of spotted seal pups in captive environments can lead to anorexia and death in severe cases. The downregulation of proteins associated with phagocytosis and proteolysis detected in the present study was consistent with high incidence of disease in captive pups. Therefore, it is necessary to improve the immunity of captive spotted seal pups through food additives or other methods. In addition, while proteomes of spotted seal adults were not measured currently, the results of spotted seal pups in this study are likely to differ from the adults. The data described here only provide preliminary insights into the physiology of this species, specifically in pups, but are by no means a comprehensive explanation of the impact of captivity on ringed seal physiology. In conclusion, the information provided herein not only expands the understanding of protein expression profiles in spotted seals, but also provides information for the conservation of this species.

## Methods

The wild *P. largha* pups investigated in this study were sampled from Liaodong Bay, China, during the Spotted seal Rescue Survey in 2019. Based on the white lanugo on their skin, and the fact that they were independent of their mother indicated that they ended lactation, but were less than one month of age. The captive *P. largha* pups used in this study were from the Dalian Sun Asia Aquarium (DSAA), China, and were all newborn under human care in 2019. The captive pups were weaned and were regularly fed prior to sampling. The diet of the captive spotted seals was a 1:1 ratio of capelin *Mallotus villosus* and Atlantic herring, *Clupea harengus* (m:m). The amount of feed was approximately 8% of the pup’s weight per day. The ontogenetic differences of wild and captive *P. largha* pups are provided in Table 4. Approximately 3 mL of blood was collected from the veins in the hind flippers when the animals were restrained on a V-shaped bench. The handling protocol was the same for wild and captive pups. Blood samples were stored in medical biochemical tubes with anti-coagulation gel at − 80 °C prior to protein extraction. All operations were performed in accordance with the relevant guidelines and regulations.

**Table 4:** The ontogenetic differences between wild and captive *P. largha* pups used in this study.

Sample ID	Birth/rescue date	Sampling date	Body weight (kg)	Body size (cm)	Age (months)	Gender	Captive/discovery location
Captive1	2019.02.19	2019.06.15	39.6	105	4	Male	DSAA, China
Captive2	2019.02.23	2019.06.15	43.5	105	4	Female	DSAA, China
Captive3	2019.02.13	2019.06.15	46.8	108	4	Male	DSAA, China
Wild1	2019.02.11	2019.02.14	12.0	75	< 1	Male	Dalian, China
Wild2	2019.02.11	2019.02.14	11.2	80	< 1	Female	Dalian, China
Wild3	2019.03.04	2019.03.05	13.5	81	< 1	Female	Panjin, China

Label-free proteomics technology was used to generate the preliminary whole blood protein expression profiles of wild and captive *P. largha* pups. Proteins from the whole blood samples were extracted using the Mammalian Tissue and Cell Protein Extraction Kit (Bangfei Bioscience Co., Ltd, Beijing, China). The concentrations of protein in each sample were determined using a protein quantification kit (Dingguo Changsheng, Beijing, China), according to the manufacturer's instructions. The extracted proteins were then excised from the preparative tube and destained with NH_4_HCO_3_. Following reduction and alkylation with dl-dithiothreitol and iodoacetamide, respectively, the samples were digested with trypsin using the Filter Aided Sample Preparation protocol^46^. All digested peptide samples were stored at − 80 °C prior to MS analysis.

MS analysis was performed according to a previous study with only one technical replicate for each sample^47^. Digested peptide mixtures were first pressure-loaded onto a fused silica capillary column packed with 3 μm dionex C18 material (Phenomenex, USA). The column was washed with buffer A (water, 0.1% formic acid) and buffer B (acetonitrile, 0.1% formic acid), and an Agilent 1100 quaternary high-performance liquid chromatography (HPLC) was applied to analyzed. The first step of HPLC measure was consisted of a 5 min gradient from 0 to 2% buffer B, followed by a 45 min gradient to 40% buffer B. Following this, a 3 min gradient from 40 to 80% buffer B was performed followed by a 10 min hold at 80% buffer B. A 2 min gradient of buffer B from 80 to 2% was performed, and approximately 100 μg of the tryptic peptide mixture was loaded onto the columns and was separated at a flow rate of 0.5 μL/min using a linear gradient. As peptides were eluted from the micro-capillary column, they were electrosprayed directly into a micrOTOF-Q II mass spectrometer (BRUKER Scientific, Beijing, China) with the application of a distal 180 °C source temperature. The mass spectrometer was operated in the MS/MS mode. Survey MS scans were acquired in the TOF-Q II with the resolution set to a value of 20,000. Each survey scan (50–2500) was followed by five data-dependent tandem mass scans at a normalized scan speed of 2 Hz.

Tandem mass spectra were searched against the reference transcriptome of spotted seals (NCBI Sequence Read Archive: SRA050171)^1^ using the Proteome Discoverer 2.1 software by the Mascot search engine (Matrix Science, London, UK; version 2.3.02) based on the standard LFQ module. The following options were used to identify the proteins: Peptide mass tolerance =  ± 15 ppm, MS/MS tolerance = 0.02 Da, enzyme = trypsin, missed cleavage = 2, fixed modification: carbamidomethyl, variable modification: oxidation, database pattern = decoy. The results were then filtered using a cutoff of 0.01 for the false discovery rate. The minimum number of peptides required to identify a protein was set to 1 and proteins with at least two unique peptides were used for abundance quantification. The quantification of peptides was based on MS1-level data. For differential expression analysis, proteins with missing abundance values in more than one biological replicate of wild or captive groups were filtered out. If a protein with more than one abundance values in one groups and missing abundance values in all three replicates in another group, it was identified as unique in this group. Then, remaining proteins with fold changes > 1.5 and p-values < 0.05 (t-test) between the wild and captive *P. largha* pups were considered to be significantly differentially expressed.

To determine the biological functions of the preliminary whole blood proteome of *P. largha* pups, the GO and KEGG annotations of the identified proteins were extracted from the annotation results of the reference transcriptome. Such annotations were obtained from our previous study by aligning the unigenes to the GO and KEGG databases^48^ using BLASTx with an E-value cutoff of less than 10^–5^^1^.

PLS-DA based on the identified proteins and their expression levels in all samples was performed using the “mixOmics” package v6.12.1^49^ in the R v4.0.0 platform to evaluate the differences in whole blood protein expression profiles between the wild and captive *P. largha* pups. The GO and KEGG enrichment analyses were performed using the DAVID tool based on the GO and KEGG annotations of DEPs^50^, and the p-values were adjusted using the FDR method. STRING^20^ was used to construct the PPI networks of the DEPs to identify the key proteins that changed the physiological functions of *P. largha* due to captivity. All other charts were drawn using the “ggplot2” package in R v4.0.0 software.

### Ethic approval

The protocols for samples collection of spotted seals was/were approved by the Ministry of Agriculture and Rural Affairs of the People’s Republic of China, permit number: 1376.

## Supplementary information

Supplementary Information 1.

Supplementary Information 2.

Supplementary Information 3.

## Data Availability

The mass spectrometry proteomics data have been deposited to the ProteomeXchange Consortium via the PRIDE partner repository with the dataset identifier PXD020112.
