# Supplementary material for: Proteomics reveals the preliminary physiological states of the spotted seal (Phoca largha) pups
Source: Sci Rep. 2020 Oct 30;10:18727. doi: 10.1038/s41598-020-75759-2 (PMC7599241; doi:10.1038/s41598-020-75759-2)
Supplement: Supplementary file 1 — Supplementary Information 1. [file 41598_2020_75759_MOESM1_ESM.docx]

### Supporting Information for

### Proteomics reveals the preliminary physiological states of the spotted seal (*Phoca largha*) pups

Jiashen Tian^1^, Jing Du^1^, Jiabo Han^1^, Xiangbo Bao^1^, Xinran Song^2^, Zhichuang Lu^1,^*

1 Dalian Key Laboratory of Conservation Biology for Endangered Marine mammals, Liaoning Ocean and Fisheries Science Research Institute, 50 Heishijiao Street, Shahekou District, Dalian, China, 116023

2 Dalian Sun Asia Tourism Holding Co., Ltd., 608-6-8 Zhongshan Road, Shahekou District, Dalian, China, 116023

*Correspondence information: [luzhichuang@hotmail.com](mailto:luzhichuang@hotmail.com)

Number files: 2 excel file (Supplementary file 1 and 2).

**Legend of supplementary tables**

**Supplementary file 1.** Information of protein identification.

**Supplementary file 2.** Annotation and quantification of identified proteins.
